# Supplementary material for: Using machine learning to determine the nationalities of the fastest 100-mile ultra-marathoners and identify top racing events
Source: PLoS One. 2024 Aug 22;19(8):e0303960. doi: 10.1371/journal.pone.0303960 (PMC11340887; doi:10.1371/journal.pone.0303960)
Supplement: Attachment — Submitted filename: PONE-D-23-43701_Revision 1_Comments for Reviewers.docx [file pone.0303960.s003.docx]

Reviewers' comments:

Reviewer's Responses to Questions

**Comments to the Author**

1. Is the manuscript technically sound, and do the data support the conclusions?

The manuscript must describe a technically sound piece of scientific research with data that supports the conclusions. Experiments must have been conducted rigorously, with appropriate controls, replication, and sample sizes. The conclusions must be drawn appropriately based on the data presented.

Reviewer #1: Yes

Reviewer #2: Partly

2. Has the statistical analysis been performed appropriately and rigorously?

Reviewer #1: Yes

Reviewer #2: N/A

3. Have the authors made all data underlying the findings in their manuscript fully available?

The [PLOS Data policy](http://www.plosone.org/static/policies.action#sharing) requires authors to make all data underlying the findings described in their manuscript fully available without restriction, with rare exception (please refer to the Data Availability Statement in the manuscript PDF file). The data should be provided as part of the manuscript or its supporting information, or deposited to a public repository. For example, in addition to summary statistics, the data points behind means, medians and variance measures should be available. If there are restrictions on publicly sharing data—e.g. participant privacy or use of data from a third party—those must be specified.

Reviewer #1: Yes

Reviewer #2: Yes

4. Is the manuscript presented in an intelligible fashion and written in standard English?

PLOS ONE does not copyedit accepted manuscripts, so the language in submitted articles must be clear, correct, and unambiguous. Any typographical or grammatical errors should be corrected at revision, so please note any specific errors here.

Reviewer #1: Yes

Reviewer #2: Yes

5. Review Comments to the Author

Please use the space provided to explain your answers to the questions above. You may also include additional comments for the author, including concerns about dual publication, research ethics, or publication ethics. (Please upload your review as an attachment if it exceeds 20,000 characters)

Reviewer #1: Manuscript Number: PONE-D-23-43701
Manuscript Title: Using Machine Learning to Determine the Nationalities of the Fastest 100-Mile Ultra-Marathoners and Identify Top Racing Events

The manuscript is interesting and well written with several strengths.
I just have a few minor comments to make.

- Overall, the different sections of the manuscript are well written.

- Line 50: delete “(ML)”.

**Answer:** We agree with the expert reviewer and deleted as suggested.

- Line 60: Please, change “offered” to “occured”.

**Answer:** We agree with the expert reviewer and changed as suggested

- Lines 79 – 80: Missed reference.

**Answer:** We agree with the expert reviewer and added as suggested

- Lines 103 – 105: Change “In this study, therefore, we sought to determine the country of origin of the fastest runners and the location of the fastest race courses.” to “In this context, we undertook this research to determine the country of origin of the fastest runners and the location of the fastest race courses.”

**Answer:** We agree with the expert reviewer and changed as suggested

- Line 142: Change “ML” to “machine learning (ML)”

**Answer:** We agree with the expert reviewer and changed as suggested

- Line 165: What is “MAE”

**Answer:** We agree with the expert reviewer and changed to MAE (Mean Absolute Error)


- Line 257 - 269: The first paragraph of the discussion is too large, I would suggest creating a small paragraph.

**Answer:** We agree with the expert reviewer and changed as suggested to: The present study aimed to determine the country of origin of the fastest 100-mile runners and the countries hosting the fastest 100-mile race courses using an XG Boost regression model. We found that the event location (*i.e.* the country where the race is held) was the most important predictor for a fast 100-mile race time where the fastest race courses are offered in Romania, Israel, Switzerland, Finland, Russia, the Netherlands, France, Denmark, Czechia, and Taiwan. Regarding the first aim, the fastest athletes come mostly from Eastern European countries (*i.e.,* Lithuania, Latvia, Ukraine, Finland, Russia, Hungary, and Slovakia).

- Line 258 – 260: Replace “Based on a recent study, we assumed that the fastest athletes would be found to originate from Sweden, Hungary, Russia, Brazil, or Lithuania” to “Based on a recent study, we assumed that the fastest athletes would come from Sweden, Hungary, Russia, Brazil, or Lithuania.”
**Answer:** since we had to reduce that section this sentence was deleted

- Line 374: delete “had”.

**Answer:** We agree with the expert reviewer and deleted as suggested.


- Line 376: delete “Better”.

**Answer:** We agree with the expert reviewer and deleted as suggested.


- Line 384: Change “offered” to “occurred”.

**Answer:** We agree with the expert reviewer and changed as suggested.


- The limitations are properly discussed.

**Answer:** no changes are required


- Figures 3, 4, 5, and 6: change “PDP” to “partial dependence plots (PDP)”.

**Answer:** We agree with the expert reviewer and changed as suggested.

Reviewer #2: The work represents an interesting attempt to apply the XGBoost algorithm to determine the country of origin of the fastest runners and to identify the fastest races. The input characteristics under consideration are: athletes nationality, age group, gender, as well as the event country, while the output is the estimation of speed. The mentioned application has several limitations, the most important of which is the selection of input characteristics, i.e., the ability to obtain a good prediction from them. Another limitation is the dataset, which contains data from different types of races (championships, recreational races), undoubtedly resulting in a biased estimator. Additionally, for certain countries, there is a very small number of samples, and samples are with a high standard deviation. All of this does not lead to a high-quality estimator. However, my recommendation is for the work to be published, considering that the authors are aware of all the shortcomings of the estimator they have made, they have adequately addressed them in the paper and addressed potential risks, so the work can be a good guide for future research in this area.
I suggest incorporating an analysis of the median as it often provides a more robust measure than the mean, particularly when dealing with datasets containing significant deviations in extreme values.

**Answer:** We recognize the limitations of this analysis, but could only use the data available. As a predictive model, there is no question about our XGB model ability (R2=0.23 in-sample tested). So the main strength of our study resides in using the model interpretability tools (reverse engineering) such as the PDP charts and the prediction distribution charts, to understand the 23% of explained output variability. Interestingly, but not as a surprise, the XGB model mostly learnt the descriptive statistical structure of the dataset.

On the comment about the median, I would like to emphasize that there is indeed a mix of mean and median values in the charts. So I´ll explain here in detail, so there are no doubts about this.

The country ranking tables (sorted by number of race records) are created simply by aggregating race records and then calculating the different stats. So they show the basic descriptive statistics (mean, std, min and max and the counts).

The PDP charts are produced after the model has been trained and represent the relative value of the model output for a predictor. The curve highlights the mean values.

Finally, the called “model interpretability charts” show group average values (means) in the middle chart (red curve) and median values in the top chart (boxplot with blue labels). Actually they are nearly the same.
